# Bodyweight and Combined Training Reduce Chronic Low-Grade Inflammation and Improve Functional Fitness of Postmenopausal Women

**DOI:** 10.3390/sports10100143

**Published:** 2022-09-23

**Authors:** Marcos Raphael Pereira Monteiro, José Carlos Aragão-Santos, Alan Bruno Silva Vasconcelos, Antônio Gomes de Resende-Neto, Leury Max da Silva Chaves, Alan Pantoja Cardoso, Albernon Costa Nogueira, Angel Carnero-Diaz, Pablo Jorge Marcos-Pardo, Cristiane Bani Corrêa, Tatiana Rodrigues de Moura, Marzo Edir Da Silva-Grigoletto

**Affiliations:** 1 Department of Physiology, Federal University of Sergipe, Aracaju 49100-000, Brazil; 2 Department of Physiotherapy, Federal University of Sergipe, Lagarto 49400-000, Brazil; 3 Department of Medicine, Federal University of Sergipe, Aracaju 49100-000, Brazil; 4 Department of Physical Education, Federal University of Sergipe, Aracaju 49100-000, Brazil; 5 Universidad Pablo de Olavide, 41013 Seville, Spain; 6 Centro Universitario San Isidoro, 41092 Seville, Spain; 7 Department of Education, Faculty of Education Sciences, University of Almería, 04120 Almería, Spain; 8 Active Aging, Exercise and Health/HEALTHY-AGE Network, Consejo Superior de Deportes (CSD), Ministry of Culture and Sport of Spain, 28040 Madrid, Spain

**Keywords:** aging, activities of daily living, inflammation, physical exercise

## Abstract

Exercise is an important tool against the deleterious effects of aging. Among the possibilities of exercise, bodyweight training (BWT) has been highlighted in the last years as a safe option to improve the health of older people. We compared the effects of 24 weeks of BWT and combined training (CT) on low-grade systematic inflammation and functional fitness in postmenopausal women. For this, 40 women were allocated and submitted to CT (n = 20, 64.43 ± 3.13 years, 29.56 ± 4.80 kg/m²) and BWT (n = 20, 65.10 ± 4.86 years, 28.76 ± 4.26 kg/m²). We measured inflammation by the interleukin-6 (IL-6), interleukin-10 (IL-10), and tumor necrosis factor-α (TNF-α) assessments. For functional fitness, we used tests similar to activities of daily living. At the end of the 16 weeks, data from 24 women were analyzed, CT (n = 14) and BT (n = 10). Both groups reduced TNF-α and IL-6 levels, without differences in IL-10. Regarding functional fitness, both groups demonstrated improvements in all tests after 24 weeks, except for rise from prone position and the 400-meter walk test for CT. In summary, CT and BWT are effective in reducing the plasma concentration of pro-inflammatory cytokines and improving functional fitness in postmenopausal women.

## 1. Introduction

Aging, associated with physical inactivity, causes a deleterious process that involves a reduction in functionality, closely related to the capacity to make activities of daily living and alteration in inflammatory factors, associated with the development of chronic diseases [1,2]. In this view, physical exercise has been presented as one of the main tools to combat this deleterious process [3,4]. In particular, for the female public, this process is increased by menopause, characterized by the clinical status after the cessation of menses for 12 months, defining the final menstrual period [5]. Amongst its various beneficial effects, physical exercise can reduce pro-inflammatory markers, such as interleukin 6 (IL-6) and tumor necrosis factor-α (TNF-α) [6].

Among the different types of physical exercise recommended for older people, we can highlight the combined training (CT). This kind of training uses strength training simultaneously with endurance training in the same session, aiming to promote improvements in the cardiorespiratory and neuromuscular systems [4]. In addition, it requires an infrastructure prepared with weight machines to perform the training, especially resistance exercises. This type of training can be applied in different ways, changing the order between aerobic and resistance exercises, therefore, promoting an interference effect between both or not [7]. In this sense, the scientific literature shows effects of CT on muscle strength, body composition, and inflammatory aspects in older women [8,9,10]. Furthermore, CT principles are aligned with the requirements of the current literature to promote multisystemic adaptations in aging, improving the ability to perform activities of daily living [1,3].

On the other hand, the bodyweight training (BWT) emerged as an alternative training method that allows the use of exercises without implements to promote health in aging. The characteristics of this method are the realization of physical exercises using body weight as only overload with no or few implements [11], being a safe option for older women [12,13,14]. This training method uses basic movement patterns, such as squat, pull, and push, similarly to the activities of daily living and promoting gains in coordination, an important factor in preventing falls in older people [15,16]. The BWT also stimulate strength and cardiorespiratory capacity [12]. In this regard, it is pertinent to investigate whether training without external overload would have similar or superior effects to training that uses implements in variables related to inflammatory aspects and activities of daily living.

Thus, our main goal was to analyze the effects of 24 weeks of bodyweight training, compared to combined training on measures of inflammatory cytokines and functional fitness in postmenopausal women. Our initial hypothesis was that bodyweight training would promote benefits similar to those found with combined training on functional fitness and inflammatory cytokines in postmenopausal women, submitted to a period of 24 weeks of training.

## 2. Materials and Methods

### 2.1. Experimental Design

**Figure 1:**
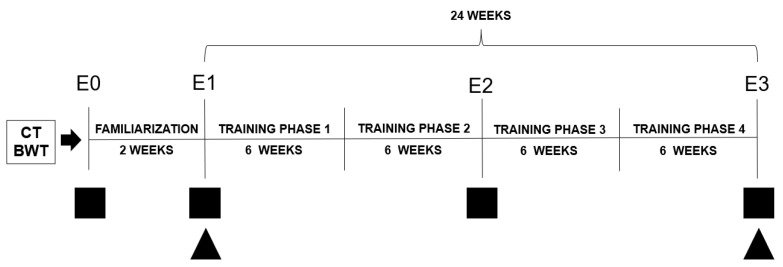
Experimental study design. E0: Evaluation pre-familiarization. E1: Evaluation pre-intervention. E2: Evaluation after 12 weeks. E3: Evaluation after 24 weeks. Black square: functional fitness evaluation. Black triangle: pro-inflammatory cytokines evaluation.

This was a longitudinal study in which 24 postmenopausal women were allocated to CT or BWT (independent variables) and underwent two weeks of familiarization and 24 weeks of training. After this, they evaluated the effects of such interventions on functional fitness and inflammation (dependent variables). For this, three evaluations were made: pre-familiarization (E0), pre-intervention (E1), after 12 weeks (E2), and after 24 weeks of intervention (E3) (Figure 1). At E0, we calculated the reproducibility of measures.

### 2.2. Subjects

Through publicity around the university and social media, we have been able to attract the interest of 50 postmenopausal women. Ten were excluded for not meeting the inclusion criteria, which were: being female, being sedentary according to the Physical Activity Questionnaire [17], not having menstrual bleeding in the last 12 months, not having cognitive disorders that compromise the understanding of the instructions and procedures, and not having a history of heart attack, uncontrolled high blood pressure, or medical contraindication for physical exercise.

**Figure 2:**
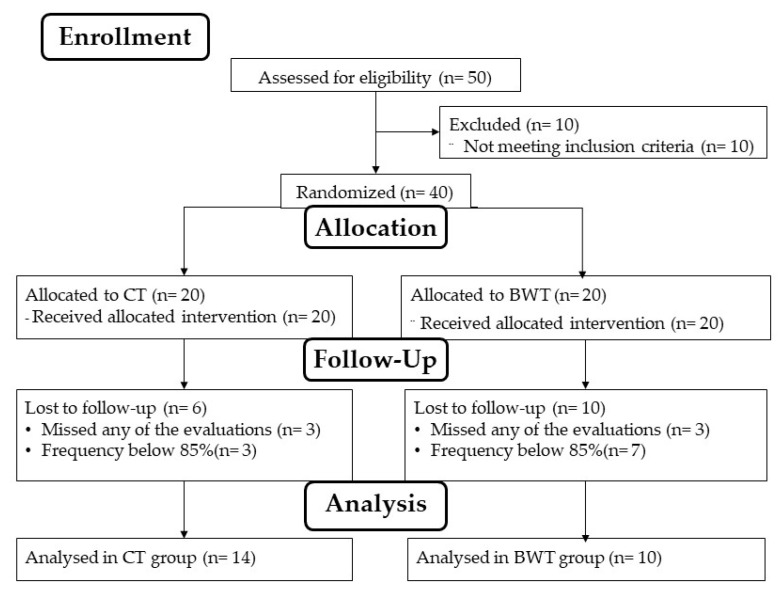
Flow diagram.

Thus, our sample was randomized in a part design, with parts of size three to distribute equally 40 active older women according to their lower limb power in the following groups: CT (n = 20) or BWT (n = 20). We organized the data from the best to the worst value and made the distribution of the groups in the same direction. An independent researcher performed the allocation procedures. The purpose of this type of distribution was to guarantee the homogeneity of the groups. The exclusion criteria adopted were: having attendance below 85% or not being present at any of the evaluation moments (Figure 2). 

We asked the participants to maintain their usual activities, not to perform any other exercise program, and not to change their eating habits. We informed the participants about all the possible risks and benefits derived from the study. In case of an agreement, we asked them to sign an informed consent form. This study was conducted following the Declaration of Helsinki for studies with human subjects, approved by the Ethical Research Committee of the Federal University of Sergipe (CAAE: 96105118.6.0000.5546), and registered in the Brazilian Clinical Trials Registry (ReBEC) as RBR-89KCHG.

### 2.3. Intervention

Both experimental groups (CT and BWT) underwent two weeks of familiarization and 72 training sessions. The sessions lasted an average of 45 min, three times a week, on non-consecutive days (Monday, Wednesday, and Friday) between 6 and 7 am, in the sports gymnasium of the institution, conducted by physical education professionals with two years previous experience. Each training session was divided into four parts, with two similar parts in both groups: Part 1, at the beginning of the session, which consists of five minutes of mobility for the main joints of the body (cervical, shoulder, hip, and ankle) and a global warm-up of ten free squats and ten jumping jacks; and Part 4, at the end of the training session, which consists of five minutes of intermittent running.

Parts 2 and 3 presented different exercises according to the specificity of each group. The CT group performed exercises commonly used in weight rooms. In Part 2, CT executed 12 min of continuous walking with a change of direction, developing cardiovascular endurance and speed. In Part 3, CT performed 16 min of resistance exercises at maximum concentric speed (variations of supine, leg extension or leg press, rowing, deadlift, lat pull down, calf raise, shoulder press, and bilateral bridge or stiff), aiming to develop strength and muscle power for upper and lower limbs in a circuit format. More details regarding the training are in the Supplementary Material (Table S1).

The BWT group predominantly has used multi-articular exercises specific for daily activities and executed with the bodyweight as only overload. Specifically, in Part 2, BWT performed 12 min of intermittent activities that stimulated the components of agility, speed, motor coordination, balance, and muscle power. In Part 3, BWT performed 16 min of multi-articular and multiplanar exercises executed at maximum concentric speed (variations of pull up, plank, dynamic quadruped exercise, bridge, lunge, push-up, and goblet squat), seeking to develop strength and muscle power for the upper and lower limbs and with high recruitment of the body mid-zone region. Both parts were performed in a circuit format. More details regarding the training are in the Supplementary Material (Table S2)

The training sessions were collective. All participants were supervised by at least two professionals specialized in physical training with a previous experience of at least two years with older people training. This ensured that the protocols were executed properly, maintaining the safety, efficiency, and motivation of the participants.

Control of the training was performed through a range of maximum repetitions, using a range of 8 to 12 repetitions [18]. For the CT group, we increase the load by 2% to 5% for upper limbs exercises, and 5% to 10% for lower limbs exercises to maintain the pre-established range of repetitions when necessary [1]. For the BWT group, when necessary, we made biomechanical adjustments or increase the complexity of the exercises to maintain the pre-established range of repetitions [19]. We controlled the effort using the OMNI-GSE scale [20], keeping Part 2 with a score between 6 and 8, and Part 3 with a score between 7 and 9. In Part 4, we maintained a score between 7 and 8. We carry out increases in exercise complexity and training density at each training phase (six weeks). The training protocol is described and available as supplementary material.

### 2.4. Data Collection

The evaluators were blinded concerning the groups of participants. In all evaluation moments, the tests were applied by the same evaluators. Physical education professionals with previous experience of at least two years performed the functional tests in the sports gymnasium of the institution. In addition, the evaluator provided verbal encouragement on all performance tests. In the week previously to familiarization, we applied the same physical tests on three different days (Monday, Wednesday, and Friday). The intraclass correlation coefficient (ICC) between days was calculated and the values were: hand grip test (HGT) (r = 0.93); five times sit to stand test (5XSTS) (r = 0.90); stand-up and walk around the house (SUWAH) (r = 0.94); rise from prone position (RPP) (r = 0.95); gallon-jug shelf-transfer (GJST) (r = 0.96); test of 400-meter walk (400 MW) (r = 0.94), suggesting excellent reliability; and dressing on and taking off a t-shirt (DTOT) (r = 0.81), suggesting good reliability. All the values were >0.7, considered sufficient to determine the reliability of a test [21].

Nursing professionals with previous experience of at least two years conducted the blood collection in the university’s physiology laboratory, with the temperature kept at 25° and relative humidity of the air kept at 60%. We requested that the sample did not perform intense exercises in the 120 h before evaluation. We asked the sample to maintain their physical activities and nutritional habits throughout all the intervention and evaluation moments. All participants were instructed to wear comfortable sports clothing at all collection moments.

#### 2.4.1. Cytokines

For biochemical evaluations, whole blood was collected, and the serum obtained was kept frozen at −80 °C until thawed for the assessment of the immune mediators. Cytokine concentrations were assessed by a multiplexed flow cytometry method using one set of bead-based immunoassays, known as the Human TH1/TH2 CBA cytokine kit II manufactured by the BD Biosciences^®^ (San Diego, CA, USA). The measurements performed followed the manufacturer’s protocols, evaluating the following circulating mediators: IL-6, IL-10, and TNF-α. 

Briefly, the lyophilized cytokine standards and the serum samples were processed. The results were acquired using the BD FACSCalibur flow cytometer, FL4 channel. Three hundred events were acquired for each cytokine bead used. Data were analyzed using the FCAP software, version 3.0 (BD Biosciences^®^, San Diego, CA, USA). Standard curves for each cytokine were generated using a standard mixture of mediators supplied. The concentration in each serum was determined by interpolation from the corresponding standard curve. Whenever a given cytokine was assessed by both kits, the mean value obtained was considered.

#### 2.4.2. Hand Grip Test 

The hand grip test evaluates the handgrip strength, using a hand dynamometer (Hand Grip Test-Jamar Plus+^®^, Bolingbrook, IL, USA). The participant realized a familiarization, followed by three valid five-second attempts, employing maximum voluntary contraction, executed at normal speed and with a gradual increase in strength. The result was computed as the average between the highest values obtained on each hand [9].

#### 2.4.3. Five Times Sit to Stand Test 

This test evaluates the functionality and, indirectly, the power of lower limbs through the individual’s ability to sit and get up from a chair. We instructed the subjects to perform five consecutive movements to sit and get up from a chair, without the help of upper limbs, as quickly as possible. The punctuation of the test is the time necessary to perform all the five repetitions, starting the movement at the command (“now”) given by the evaluator. Three attempts were performed as familiarization. Thirty seconds of rest were given between each execution. In the end, all attempts were timed and recorded, and finally, the shortest time was used for analysis [22].

#### 2.4.4. Stand-Up and Walk around the House 

The stand-up and walk-around-the-house test (SUWAH) evaluate the agility and dynamic balance of the individual, using the functional movement of getting up from a sitting position and walking a certain distance with some changes in direction. Initially, a chair was positioned with two cones at a distance of four meters backward and three meters sideways, a cone on each side. The test is characterized by performing a route involving walking to both cones and returning to the chair twice, in the shortest possible time. Each participant performed the test three times. Between each execution, the participant had a recovery of 60 s. We analyzed the shortest time [23,24].

#### 2.4.5. Rise from Prone Position 

This test consists in globally evaluating the individual’s autonomy. For this, the individual is initially positioned in a prone position on a mat on the floor and instructed to take the bipedal position as quickly as possible as soon as given the command “now”. The execution of the test is timed and was performed three times. The first of which was for familiarization purposes, with a 30-s interval between each execution. The shortest time achieved was forwarded for further analysis [24].

#### 2.4.6. Dressing on and Taking off a T-Shirt 

The purpose of this test is to evaluate the functional autonomy related to the movement of the upper limbs, involving mobility, speed, and coordination. The subject begins the test in an orthostatic position, with arms relaxed along the body and a shirt in the dominant hand. At the command “now” the subject must put on the shirt and immediately remove it, as quickly as possible, returning to the initial position. A familiarization was performed followed by two attempts. The participants had thirty seconds of recovery between each execution. The shortest time was analyzed [24].

#### 2.4.7. Gallon-Jug Shelf-Transfer 

The purpose of this test is to evaluate the individual’s physical function globally, involving aspects of core stability, strength, coordination of upper and lower limbs, and handgrip strength. The test begins by positioning the subject laterally to a bookcase, with five bottles positioned side-by-side on the bottom shelf.

We instructed the participant to sequentially transfer five gallons of liquid weighing about 3.9 kg, from a lower shelf to an upper shelf, as quickly as possible. For this, the participant needs to perform small squats and trunk rotation to reach the lower shelf and move all the bottles to the upper shelf. The participant needs to move one bottle at a time and use both hands. We record the time to complete the task, starting with the command provided by the evaluator (“now”) and ending with the proper positioning of the last bottle on the top shelf. Between each attempt, the participant had 60 s of recovery. We instructed a familiarization trial followed by two attempts recorded. The shortest time was used for the analyses [25].

#### 2.4.8. Test of 400-Meter Walk

In this test, each subject was instructed to walk 20 laps as fast as possible, without running, on a previously delimited 20-m course. Only one attempt was made. We record the time for analysis [26].

### 2.5. Statistical Analysis

We performed a power analysis to determine the appropriate number of participants to detect a minimum difference of 10% in IL-6 and TNF-α, with a power of 80%. The sample required was ten subjects. We expressed the data in descriptive statistics with mean and standard deviation. In the sample characterization, we used independent test T for numerical values and the chi-square test for categorical values. Initially, we did a verification of normality through the Shapiro–Wilk test. The homogeneity of variances was verified through Levene’s test. After this, we analyzed the data using repeated-measures analysis of variance (ANOVA), and when *p* < 0.05, we realized multiple comparisons with Bonferroni correction; additionally, partial η² was calculated for the interactions. For non-normal and heterogeneous data, the Wilcoxon test was used for analysis over time, and the Mann–Whitney U-test for between-group analysis. In addition, we used Cohen’s D to measure the effect size of the comparisons performed [27]. All analyses were made using Jamovi software 2.2.5 [28].

## 3. Results

Firstly, the sample evaluated in this study presented baseline values with no statistical differences in their characterization (Table 1); similarly, no statistically significant differences were found between the groups in the initial moment for any of the outcome variables.

**Figure 3:**
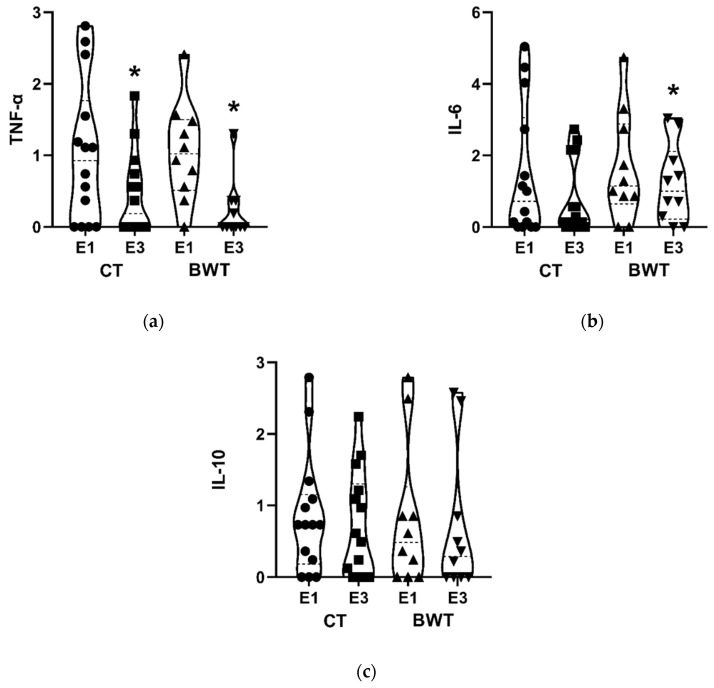
Effects of 24 weeks of combined training (CT) and bodyweight training (BWT) on cytokine concentrations of postmenopausal women. E1: Evaluation pre-intervention; E2: Evaluation after 12 weeks. E3: Evaluation after 24 weeks. * Difference to the E1 moment: Circle: Sample values at E1 of CT. Square: Sample values at E3 of CT. Triangle: Sample values at E1 of BWT. Inverse triangle: Sample values at E3 of BWT. (**a**) Effects of intervention on TNF-α; (**b**) Effects of intervention on IL-6; (**c**) Effects of intervention on IL-10.

When analyzing cytokine plasma concentrations (Figure 3), there was no effect in the comparison between groups. However, significant reductions were observed for TNF-α concentrations over time for both groups (CT: d = 0.778; BWT: d = 0.944). In turn, for IL-6 concentrations, this difference occurred only for the BWT group (d = 0.400).

Regarding the intervention effects on the functional parameters (Table 2), variance analysis showed no interaction effect between time and group for any of the variables. However, regarding time, we could observe that for the HGT, both experimental groups showed significant improvements between E1 and E3 (CT: d = 0.434; BWT: d = 0.657), and for the BWT group, there were also differences between E2 and E3 (d = 0.588).

For 5XSTS, both groups showed significant reductions between E1 and E2 (CT: d = 0.749; BWT: d = 0.827), maintaining when comparing E1 and E3 (CT: d = 1.339; BWT: d = 1.421); however, between E2 and E3, only CT showed significant differences (d = 0.503).

Regarding the SUWAH, significant reductions were observed between E1 and E3 for both groups (CT: d = 0.860; BWT: d = 1.221), but only the CT group showed reductions between E1 and E2 (d = 0.611), and only BWT between E2 and E3 (d = 0.919). As for RPP, only the BWT group showed significant reductions between E1 and E3 (d = 0.593) and E2 and E3 (d = 0.382).

For DTOT, only the CT group showed significant reductions between E1 and E2 (d = 0.556), however, both groups showed differences between the moments E1 and E3 (CT: d = 0.690; BWT: d = 0.969). For the GJST, both groups showed significant reductions between E1 and E3 (CT: d = 0.973; BWT: d = 1.124), and between the moments E2 and E3 (CT: d = 0.515; BWT: d = 0.849). Finally, for the 400 MW a significant differences were found for the BWT group between times E1 and E3 (d = 1.178).

## 4. Discussion

The main findings of this article showed that the BWT was able to promote similar improvements to the CT for postmenopausal women in the period of 24 weeks, for the majority variables of pro-inflammatory cytokines and functional fitness, partially confirming our initial hypothesis. Furthermore, it was possible to notice that BWT promoted positive adaptations in more variables analyzed than CT.

Regarding the pro-inflammatory cytokines, both training programs reduced the plasma concentration of TNF-α, however, only the BWT attenuated the levels of IL-6, denoting an important anti-inflammatory effect in this population. Possibly, this finding is related to training-induced changes in body composition. This idea is reinforced by the findings of Sardeli et al. [29] who suggest that reduced concentrations of pro-inflammatory cytokines may be closely associated with changes in body composition, especially reduced fat percentage and increased muscle mass. Although we were unable to verify measures related to body composition, a limitation of our study, the duration of the study indicates a good chance that significant adaptations in body composition occurred in addition to adaptations in strength tests involving body weight.

Despite reducing pro-inflammatory cytokines, both groups have not improved anti-inflammatory cytokine (IL-10). These findings have also been found in studies investigating other exercise modalities, such as functional training [10] and aerobic training [30]. The physiological explanation is that adaptations for anti-inflammatory cytokines seem to be dependent on variables other than exercise. Indeed, our findings corroborate Vasconcelos et al. [10] who reinforced that there were no differences in IL-10 concentration after 24 weeks of functional or combined training in elderly women. It is important to know that low-grade systemic inflammation (inflammaging) is related to the onset or progression of several chronic diseases, such as hypertension and cancer [31], denoting that both training programs are effective options for health promotion. 

Concerning handgrip strength, both protocols improved this variable. It is known that there is an important decline after 45 years of age and low values in this variable are associated with mortality and disability [32]. In this perspective, strength training is shown to be efficient to attenuate the loss of grip strength [33], an outcome also found in the present study. In CT, exercises performed on machines and with implements require the stimulation of this capacity. Interestingly, BWT was also able to improve strength values, even though it presented fewer exercises that required manual grip.

Regarding functionality, both training protocols were able to promote improvements in lower limb functionality when analyzed through 5XSST and SUWAH. The improvement in 5XSTS values is in agreement with the current literature, such as the studies by Santos et al. [12] and Aragão-Santos et al. [34], which showed improvements in the performance of this test after BWT and CT programs. This outcome was expected, since both models of training presented characteristics that would favor task performance, developing motor actions similar to those of the tests, as well as the maximum concentric speed employed in the execution of the exercises [35]. Specifically, for SUWAH, the BWT showed significant differences not found by the CT. This is possibly due to the specificity provided by the exercises with body weight, such as the change of direction present in exercises, providing a more specific transfer for the test.

Also, the participants in CT and BWT had better performances in the DTOT and GJST tests, denoting improvement in the upper limbs and global functionality. Regarding the DTOT, this outcome may be associated with the effect of exercise on motor coordination, agility, and mobility of the upper limbs. Part 1 training, which focuses on preparing muscles and joints for movement, may have contributed to these positive effects [36] in addition to the ability of resistance training to increase range of motion [37]. The GJST, on the other hand, comprises the analysis of muscle strength, agility, and global coordination [25]. Here, we observed positive adaptations after both training protocols, since performing the exercises at maximum concentric speed favors the increase in muscle power, which contributes positively to fast task execution [38].

However, only the BWT was able to provide adaptations at the level of anaerobic and aerobic endurance for the studied population when the 400 MW test was used, corroborating other investigations [39,40]. This adaptation can be explained by the neuromuscular and metabolic characteristic of the BWT session itself, composed of exercises organized in a circuit, executed mainly in a closed kinetic chain and at maximum concentric speed, which has efficient transference to daily living tasks, such as walking [41].

Among the main limitations of our study, we can point out that we did not perform the intention to treat analysis due to the high sample loss. This can be explained since we have established strict exclusion criteria related to frequency (85% of attendance) throughout 24 weeks of training, designed to keep the training at least twice a week, following the current guidelines for physical exercise for seniors [1,3]. Another limitation in our study was the absence of a control group that did not perform any intervention; however, we consider the CT as a positive control group, since it has proven efficacy in the literature on the outcomes analyzed [42,43]. Furthermore, from the ethical perspective of health interventions, we believe that leaving one group without any intervention would be ethically questionable. Therefore, we adopted the design used and analyzed the reliability of all tests aiming to minimize possible confounding factors.

To our best knowledge, the present study is the first evidence showing the effects of BWT on inflammatory and fitness parameters in postmenopausal women. The interaction of these variables may contribute to expanding the knowledge about the effects of different training methods on aging. Among our positive points, we can mention the long intervention time, the strictness of the exclusion criteria, and the promotion of applicable supplementary material for movement professionals who work with postmenopausal women. Regarding suggestions for future research, this article provides a detailed basis for the practical application of the BWT, which could possibly be used as a comparison group to different populations and in different contexts.

## 5. Conclusions and Practical Application

Twenty-four weeks of CT and BWT are effective in reducing the plasma concentration of pro-inflammatory cytokines and improving functional fitness in postmenopausal women. From a practical point of view, BWT has a low cost and is applicable in different contexts in reality by movement professionals involved with the aging population. Furthermore, this article provides a detailed basis for the practical application of two different protocols of training in a scientific and professional context for postmenopausal women. These protocols can be alternatives for professionals in the area, can be applied to other populations in future research, and may contribute to better efficiency of health promotion programs. 

## Figures and Tables

**Table 1:** Sample characterization.

Characteristics	CT (n = 14)	BWT (n = 10)	*p* Interaction
Anthropometry (mean and standard deviation)			
Age (years)	64.43 ± 3.13	65.10 ± 4.86	0.685
Body mass (kg)	71.95 ± 8.36	67.92 ± 10.14	0.298
Height (m)	1.57 ± 0.06	1.54 ± 0.06	0.258
BMI (kg/m²)	29.56 ± 4.80	28.76 ± 4.26	0.680
WHR	0.86 ± 0.10	0.88 ± 0.10	0.763
Medical History (relative and absolute frequency)			
Hypertension	35.7 (5)	50.0 (5)	0.484
Diabetes	7.1 (1)	10.0 (1)	0.803
Dyslipidemia	42.9 (6)	50.0 (5)	0.729
Osteoarthritis	28.6 (4)	20.0 (2)	0.633
Arthritis	21.4 (3)	40.0 (4)	0.324
Depression	7.1 (1)	40.0 (4)	0.051

Note: BMI: body mass index. WHR: waist–hip ratio. CT: combined training group. BWT: bodyweight training group.

**Table 2:** Effects of 24 weeks of combined training (CT) and bodyweight training (BWT) on functional fitness in menopausal women.

	E1	E2	E3	Analysis
**HGT (kg/F)**				
CT	22.46 ± 4.24	23.96 ± 3.81 (∆ = 6.67%; d = 0.372; CI = [−3.108; 0.108])	24.21 ± 3.82 * (∆ = 7.79%; d = 0.434; CI = [−3.242; −0.258])	p time < 0.001 p interaction = 0.064 η² parcial = 0.118
BWT	21.80 ± 4.72	22.35 ± 3.91 (∆ = 2.52%; d = 0.127; CI = [−2.448; 1.358])	24.70 ± 4.08 *⸸ (∆ = 13.30%; d = 0.657; CI = [4.660; −1.330])	
**5XSTS (s)**				
CT	7.75 ± 1.54	6.57 ± 1.61 * (∆ = −15.23%; d = 0.749; CI = [0.494; 1.858])	5.83 ± 1.32 *⸸ (∆ = −24.77%; d = 1.339; CI = [1.335; 2.487])	p time < 0.001 p interaction = 0.998 η² parcial = 0.000
BWT	7.77 ± 1.36	6.59 ± 1.49 * (∆ = −15.18%; d = 0.827; CI = [0.368; 1.982])	5.88 ± 1.30 * (∆ = −24.19%; d = 1.421; CI = [1.210; 2.574])	
**SUWAH (s)**				
CT	33.68 ± 3.41	31.72 ± 2.99 * (∆ = −5.82%; d = 0.611; CI = [0.009; 3.911])	30.59 ± 3.77 * (∆ = −9.17%; d = 0.860; CI = [0.802; 5.380])	p time < 0.001 p interaction = 0.732 η² parcial = 0.009
BWT	34.19 ± 3.11	32.78 ± 1.88 (∆ = −4.12%; d = 0.549; CI = [−0.798; 3.818])	30.84 ± 2.32 *⸸ (∆ = −9.79%; d = 1.221; CI = [0.645; 6.061])	
**RPP (s)**				
CT	2.56 ± 1.00	2.50 ± 0.95 (∆ = −2.34%; d = 0.062; CI = [−0.222; 0.349])	2.42 ± 0.88 (∆ = −5.49 %; d = 0.149; CI = [−0.209; 0.506])	p time < 0.001 p interaction = 0.034 η² parcial = 0.143
BWT	2.87 ± 1.21	2.60 ± 1.02 (∆ = −9.40%; d = 0.241; CI = [−0.072; 0.604])	2.22 ± 0.97 *⸸ (∆ = −22.64% d = 0.593; CI = [0.227; 1.073])	
**DTOT (s)**				
CT	13.70 ± 2.59	12.39 ± 2.10 * (∆ = −9.56%; d = 0.556; CI = [0.022; 2.611])	12.08 ± 2.08 * (∆ = −11.82%; d = 0.690; CI = [0.218; 3.028])	p time < 0.001 p interaction = 0.728 η² parcial = 0.011
BWT	14.10 ± 2.11	12.86 ± 1.43 (∆ = −8.79%; d = 0.688; CI = [−0.291; 2.773])	12.08 ± 2.06 * (∆ = −14.32%; d = 0.969; CI = [0.361; 3.685])	
**GJST (s)**				
CT	10.68 ± 0.80	10.41 ± 1.09 (∆ = −2.53%; d = 0.282; CI = [−0.265; 0.808])	9.93 ± 0.74 *⸸ (∆ = −7.02%; d = 0.973; CI = [0.330; 1.166])	p time < 0.001 p interaction = 0.631 η² parcial = 0.021
BWT	11.11 ± 0.92	10.80 ± 0.76 (∆ = −2.79%; d = 0.367; CI = [−0.323; 0.947])	10.12 ± 0.84 *⸸ (∆ = −8.91%; d = 1.124; CI = [0.495; 1.485])	
**400 MW (s)**				
CT	242.92 ± 28.82	222.57 ± 38.88 (∆ = −8.38%; d = 0.595; CI = [2.040; 38.670])	229.78 ± 31.55 (∆ = −5.41%; d = 0.435; CI = [−5.175; 31.460])	p time = 0.003 p interaction = 0.283 η² parcial = 0.056
BWT	239.70 ± 23.02	222.40 ± 23.32 (∆ = −7.22%; d = 0.747; CI = [−4.374; 38.971])	212.40 ± 21.70 * (∆ = −11.38%; d = 1.220; CI = [5.626; 31.672])	

Note: * Statistically significant difference to the E1; ⸸ Statistically significant difference to the E2 moment; ∆: Percentage change related to the comparison to the E1 moment; d: Effect size related to the comparison to the E1 moment; CI: Confidence interval for the difference related to the comparison to the E1 moment. HGT: hand grip test, 5XSTS: five times sit to stand, SUWAH: stand-up and walking around the house, RPP: rise from prone position, DTOT: dressing on and taking off a t-shirt, GJST: gallon-jug shelf-transfer, 400 MW: 400-m walk.

## Data Availability

Not applicable.

## Supplementary Materials

The following supporting information can be downloaded at: https://www.mdpi.com/article/10.3390/sports10100143/s1, Table S1: Description of the combined training sessions conducted over 24 weeks; Table S2: Description of the bodyweight training sessions conducted over 24 weeks.
