# Supplementary material for: Bodyweight and Combined Training Reduce Chronic Low-Grade Inflammation and Improve Functional Fitness of Postmenopausal Women
Source: Sports (Basel). 2022 Sep 23;10(10):143. doi: 10.3390/sports10100143 (PMC9610763; doi:10.3390/sports10100143)
Supplement: Supplementary file 1 [file sports-10-00143-s001.zip › sports-1866024-supplementary.pdf]

Table S1. Description of the combined training sessions conducted over 24 weeks.

	Part 1	Part 2		Part 3		Part 4
		Phase 1 (1-18 session)	Phase 2 (18-36 session)	Phase 1 (1-18 session)	Phase 2 (18-36 session)	
	Mobility exercises for the cervical, shoulder, hip and ankle joints<br>10 squats<br>10 jumping jacks	Interval Walking<br>(30 seconds walking alternated with 30 seconds running)	Interval Walking<br>(30 seconds walking alternated with 30 seconds running)	Vertical Supine	Horizontal Supine	Intermittent running
				Leg Extension	Leg Press 45º	
				Neutral Rowing	Pronated Rowing	
				Deadlift	Smith Squat	
				Lat Pull Down (Pronated)	Lat Pull Down (Neutral)	
				Smith bilateral calf raise	Unilateral calf raise on step	
				Shoulder press (Pronated)	Shoulder press (Neutral)	
				Stiff	Bilateral bridge	
**Combined Training**	**Total time: 5 min; 3-5 exercises per joint; 1 set of 8 seconds**	**Total time: 12 min; OMINI-GSE: 6/7**	**Total time: 12 min; OMINI-GSE: 6/7**	**Total time: 16 min; 2 sets of 8/12 repetitions; 1 minute per station; Density: 1/1; OMINI-GSE: 7/9**	**Total time: 16 min; 2 sets of 8/12 repetitions; 1 minute per station; Density: 1/1; OMINI-GSE: 7/9**	**Total time: 5 min; 5/8 times; Density: 1/1; OMINI-GSE: 7/8**
		**36-54 sessões**	**54-72 sessões**	**36-54 sessões**	**54-72 sessões**	
	Mobility exercises for the cervical, shoulder, hip and ankle joints<br>10 squats<br>10 jumping jacks	Interval Walking<br>(40 seconds walking alternated with 20 seconds running)	Interval Walking<br>(40 seconds walking alternated with 20 seconds running)	Vertical Supine	Horizontal Supine	Intermittent running
				Leg Extension	Leg Press 45º	
				Neutral Rowing	Pronated Rowing	
				Deadlift	Smith Squat	
				Lat Pull Down (Pronated)	Lat Pull Down (Neutral)	
				Bilateral calf raise on step	Unilateral calf raise on step	
				Shoulder press (Pronated)	Shoulder press (Neutral)	
				Stiff	Bilateral bridge	
	**Total time: 5 min; 3-5 exercises per joint; 1 set of 8 seconds**	**Total time: 12 min; OMINI-GSE: 6/8**	**Total time: 12 min; OMINI-GSE: 6/8**	**Total time: 16 min; 2 sets of 8/12 repetitions; 1 minute per station; Density: 1/1; OMINI-GSE: 7/9**	**Total time: 16 min; 2 sets of 8/12 repetitions; 1 minute per station; Density: 1/1; OMINI-GSE: 7/9**	**Total time: 5 min; 5/8 times; Density: 1/1; OMINI-GSE: 7/8**

Table S2. Description of the bodyweight training sessions conducted over 24 weeks

	Part 1	Part 2		Part 3		Part 4
		**Phase 1 (1-18 session)**	**Phase 2 (18-36 session)**	**Phase 1 (1-18 session)**	**Phase 2 (18-36 session)**	
**Bodyweight training**	Mobility exercises for the cervical, shoulder, hip and ankle joints<br>10 squats<br>10 jumping jacks	10-meter sprint with 5-second rest	Two 10-meter sprint with 5-second rest	Adapted pull up with neutral distance between hands (pronated)	Adapted pull up with neutral distance between hands (pronated)	Intermittent running
		Fast anterior and posterior displacement over the line marked on the floor	Fast anterior and posterior displacement over two lines 5 centimeters apart	Front plank (30 cm bench)	Front plank associated to the side plank (30 cm bench)	
				Dynamic quadruped exercise (1 limb movement)	Dynamic quadruped exercise (2 limbs movement)	
		Lateral displacement, 10-meter distance	Lateral displacement in and out over a distance of 15 meters	Bilateral bridge	Unilateral bridge	
				Adapted pull up with neutral distance between hands (pronated)	Adapted pull up with short distance between hands (pronated)	
		Five repetitions of Jumping Jack and 2 seconds pause in moderate speed	Ten repetitions of Jumping Jack and 2 seconds pause in moderate speed	Lunge	Reverse lunge	
				Push ups on 40 cm table	Push-ups on a 40 cm table with distance between hands alternating between long and short	
		Lateral raising of the upper limbs (moderate speed)	Lateral raising of the upper limbs (fast speed)	Goblet squat	Goblet squat associated with deadlift	
	**Total time: 5 min; 3-5 exercises per joint; 1 set of 8 seconds**	**Total time: 12 min; 2 times in each exercise; 1 minute and 20 seconds per station; Density: 1/1; OMINI-GSE: 6/7**	**Total time: 12 min; 2 times in each exercise; 1 minute and 20 seconds per station; Density: 1/1; OMINI-GSE: 6/7**	**Total time: 16 min; 2 sets of 8/12 repetitions; 1 minute per station; Density: 1/1; OMINI-GSE: 7/9**	**Total time: 16 min; 2 sets of 8/12 repetitions; 1 minute per station; Density: 1/1; OMINI-GSE: 7/9**	**Total time: 5 min; 5/8 times; Density: 1/1; OMINI-GSE: 7/8**
		**Phase 3 (36-54 session)**	**Phase 4 (54-72 session)**	**Phase 3 (36-54 session)**	**Phase 4 (54-72 session)**	
	Mobility exercises for the cervical, shoulder, hip and ankle joints<br>10 squats<br>10 jumping jacks	10-meter sprint with change of direction	Lateral jump on the step	Adapted pull up with distance between hands alternating between long and short (pronated)	Adapted pull up with distance between hands alternating between long and short (pronated)	Intermittent running
		Fast anterior and posterior displacement over two lines 5 centimeters apart associated with ascent and descent on a 20 cm step	Fast anterior and posterior displacement over two lines 5 centimeters apart associated with jump on a 20 cm step	Frontal plank with lateral displacement of the lower limb on the 30 cm bench	Front plank with front lower limb elevation on the 30 cm bench	
		Lateral displacement with change of direction	Lateral displacement with change of direction and coordinated movement of upper limbs	Dynamic quadruped exercise (cross patern)	Mountain Climber	
		Alternate jumping jack with stationary march at high speed	Alternate jumping jack displacement with stationary march at high speed	Unilateral bridge on a 20 cm step	Unilateral bridge on a 20 cm step alternating legs	
				Adapted pull up with short distance between hands (pronated)	Adapted pull up with short distance between hands (pronated)	
		Frontal and lateral raising of the upper limbs alternated with one leg	Frontal and lateral raising of the upper limbs alternated with one leg associated with displacement	Goblet squat associated with two-step lateral displacement	Goblet squat associated with displacement in different directions	
				Push ups on 30 cm bench	Push-up on the 30 cm bench associated with upper limb movement to touch the shoulder	
				Goblet squat associated with reverse lunge	Goblet squat at maximum speed with 2-second pause every five repetitions	
	**Total time: 5 min; 3-5 exercises per joint; 1 set of 8 seconds**	**Total time: 12 min; 2 times in each exercise; 1 minute and 20 seconds per station; Density: 2/1; OMINI-GSE: 6/8**	**Total time: 12 min; 2 times in each exercise; 1 minute and 20 seconds per station; Density: 2/1; OMINI-GSE: 6/8**	**Total time: 16 min; 2 sets of 8/12 repetitions; 1 minute per station; Density: 1/1; OMINI-GSE: 7/9**	**Total time: 16 min; 2 sets of 8/12 repetitions; 1 minute per station; Density: 1/1; OMINI-GSE: 7/9**	**Total time: 5 min; 5/8 times; Density: 1/1; OMINI-GSE: 7/8**
